# The role of pericytic laminin in blood brain barrier integrity maintenance

**DOI:** 10.1038/srep36450

**Published:** 2016-11-03

**Authors:** Jyoti Gautam, Xuanming Zhang, Yao Yao

**Affiliations:** 1 College of Pharmacy, University of Minnesota, 1110 Kirby Drive, Duluth, MN, 55812, USA.

## Abstract

Laminin, a major component of the basement membrane, plays an important role in blood brain barrier regulation. At the neurovascular unit, brain endothelial cells, astrocytes, and pericytes synthesize and deposit different laminin isoforms into the basement membrane. It has been shown that laminin α4 (endothelial laminin) regulates vascular integrity at embryonic/neonatal stage, while astrocytic laminin maintains vascular integrity in adulthood. Here, we investigate the function of pericyte-derived laminin in vascular integrity. Using a conditional knockout mouse line, we report that loss of pericytic laminin leads to hydrocephalus and BBB breakdown in a small percentage (10.7%) of the mutants. Interestingly, BBB disruption always goes hand-in-hand with hydrocephalus in these mutants, and neither symptom is observed in the rest 89.3% of the mutants. Further mechanistic studies show that reduced tight junction proteins, diminished AQP4 expression, and decreased pericyte coverage are responsible for the BBB disruption. Together, these data suggest that pericyte-derived laminin is involved in the maintenance of BBB integrity and regulation of ventricular size/development.

The blood brain barrier (BBB) is a dynamic structure that maintains the homeostasis of the central nervous system (CNS)^1^,^2^. Accumulating evidence suggests that BBB breakdown not only is a consequence of but also contributes to the pathogenesis of many neurological disorders, including Alzheimer’s disease and stroke. The BBB is composed of cellular components, including endothelial cells, pericytes, and astrocytic endfeet, and non-cellular component—the basement membrane (BM). Most BBB research focuses on its cellular components, leaving the BM understudied probably due to its intrinsic complexity. Although damage to the BM leads to BBB breakdown and intracerebral hemorrhage in pathological conditions, such as ischemic stroke^3^,^4^,^5^,^6^,^7^, the role of BM in BBB regulation in normal conditions remains largely unknown.

Laminin, a trimeric protein containing α-, β-, and γ-subunits, is a major component of the BM^8^,^9^. It has a variety of biological functions, including cell adhesion, cell differentiation, and BM assembly, which explains its important roles in embryonic development, organogenesis, and vascular integrity^8^,^9^,^10^,^11^. Recently, we have shown that laminin’s functions are dependent on its cellular origins since different cell types synthesize distinct laminin isoforms^12^. For example, brain endothelial cells make laminin-α4β1γ1 (−411) and -α5β1γ1 (−511)^13^,^14^,^15^, whereas astrocytes generate laminin-α1β1γ1 (−111) and -α2β1γ1 (−211)^15^,^16^. In addition to brain endothelial cells and astrocytes, pericytes have also been found to produce laminin^17^,^18^,^19^,^20^.

Previous study showed that mice lacking laminin α4 developed hemorrhage at embryonic/neonatal stage^21^, suggesting a critical role of laminin α4 in vascular permeability at early developmental stage. Using conditional knockout technique, we reported that astrocytic laminin-deficient mice had BBB disruption and age-dependent intracerebral hemorrhage^22^,^23^, suggesting that astrocytic laminin functions to maintain BBB integrity. The role of pericytic laminin in BBB regulation, however, is still elusive. Here we show that PDGFRβ^+^ pericyte-derived laminin is abrogated in all conditional knockout mice, but only 10.7% demonstrate CNS phenotype, including hydrocephalus and BBB breakdown. Further mechanistic studies reveal that reduced tight junction protein expression, diminished AQP4 level, and decreased pericyte coverage are responsible for the BBB disruption in the hydrocephalic mutants. These data suggest that pericytic laminin is involved in the maintenance of BBB integrity and regulation of ventricular size/development.

## Results

### 10.7% PKO mice develop hydrocephalus

**Figure 1:**
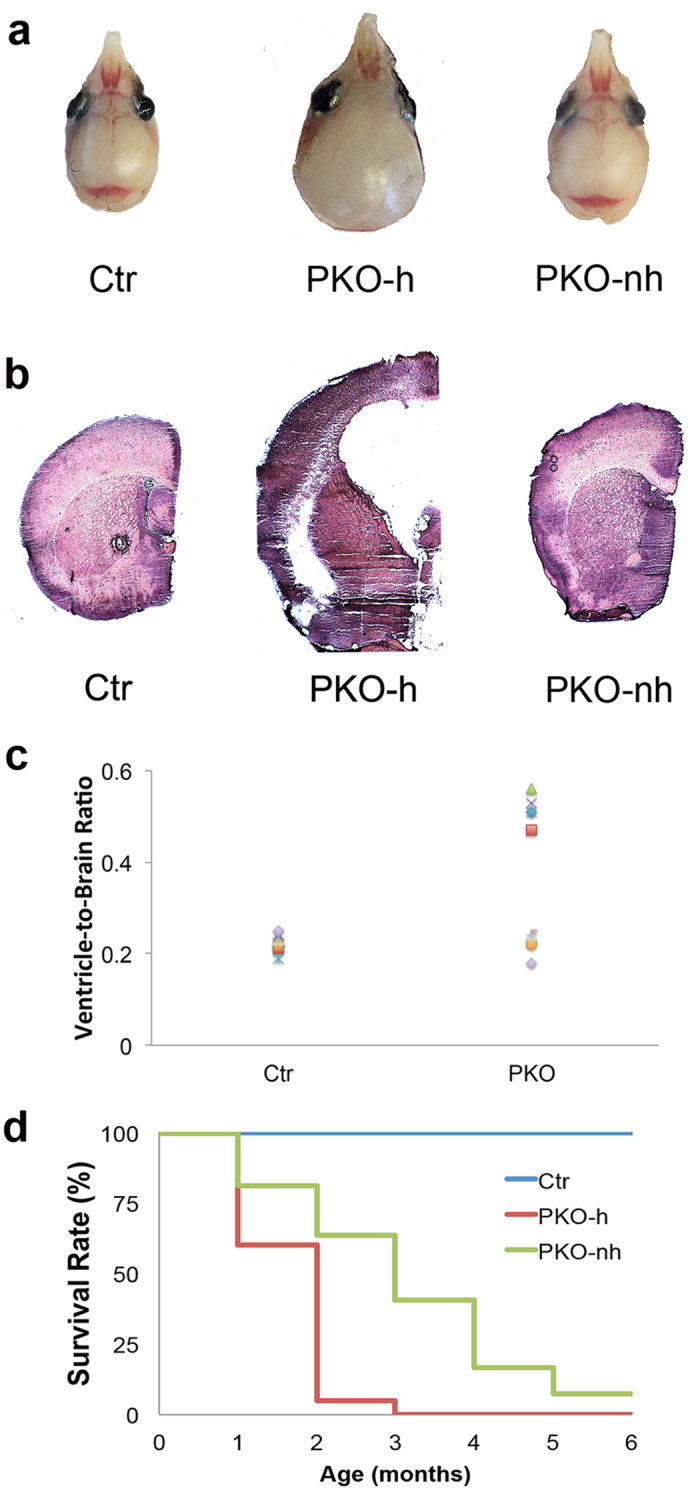
Hydrocephalic phenotype of PKO mice. (**a**) Gross brain images of 4-week-old control, PKO-h, and PKO-nh mice. (**b**) H&E staining of anterior brain sections of control, PKO-h, and PKO-nh mice. (**c**) Plot of ventricle-to-brain ratio over genotypes. (**d**) Survival rate of Ctr (blue), PKO-h (red) and PKO-nh (green) mice. Ctr, controls; PKO, laminin γ1^flox/flox^:Pdgfrβ-Cre^+^; PKO-h, PKO mice with hydrocephalus; PKO-nh, PKO mice without hydrocephalus; H&E, hematoxylin & eosin.

Although there are no pericyte-specific markers available, PDGFRβ is widely used as a marker for mural cells^24^,^25^, which include both pericytes and vascular smooth muscle cells (VSMCs). By crossing the laminin γ1^flox/flox^ mice with the Pdgfrβ-Cre^+^ line, we generated a conditional knockout mouse line with laminin deficiency in mural cells (laminin γ1^flox/flox^:Pdgfrβ-Cre^+^, named PKO hereafter). These PKO mice are born at the expected Mendelian ratio and usually die within 4 months^26^. Compared to their littermate controls (laminin γ1^flox/flox^ and/or laminin γ1^flox/+^ PDGFRβ-Cre^+^), all PKO mice develop a severe muscular dystrophic phenotype^26^. In addition to the muscle defect, we also noticed a hydrocephalic phenotype, which was usually detected 2 weeks after birth, in 10.7% of PKO mice. Representative gross brain images and H&E-stained brain sections from 4-week-old control and PKO mice are shown in Fig. 1a,b. To investigate the severity of this phenotype, we measured ventricle-to-brain ratio, a parameter that positively correlates with ventricle size^27^,^28^. Two distinct populations in PKO group were observed: one with ventricle-to-brain ratio comparable to the controls and one with larger ventricle-to-brain ratio (Fig. 1c). To differentiate them, the former is named PKO-non-hydrocephalus (PKO-nh) and the latter is named PKO-hydrocephalus (PKO-h). To determine the time course of this hydrocephalic phenotype, we analyzed these mice at two different developmental stages: Embryonic day (E) 15.5 and postnatal day (P) 2. No hydrocephalic embryos/pups were found at these time points (not shown), suggesting that this phenotype occurs at later stages. It should be noted that the hydrocephalic phenotype, unlike the muscle pathology, was not observed in all PKO mice. Statistics showed that 10.7% of the PKO mice were hydrocephalic, whereas only 0.5% of the littermate controls were hydrocephalic (Supplementary Table 1). These results suggest that PDGFRβ^+^ cell-derived laminin is involved in the formation of hydrocephalus either directly or indirectly. In addition, survival analysis has demonstrated that the PKO-h mice usually die within 2 months after birth (Fig. 1d). Although the PKO-nh mice have a longer life span (Fig. 1d), they usually die within 4 months probably due to respiratory failure caused by congenital muscular dystrophy^26^.

### PKO-h but not PKO-nh mice show BBB disruption

**Figure 2:**
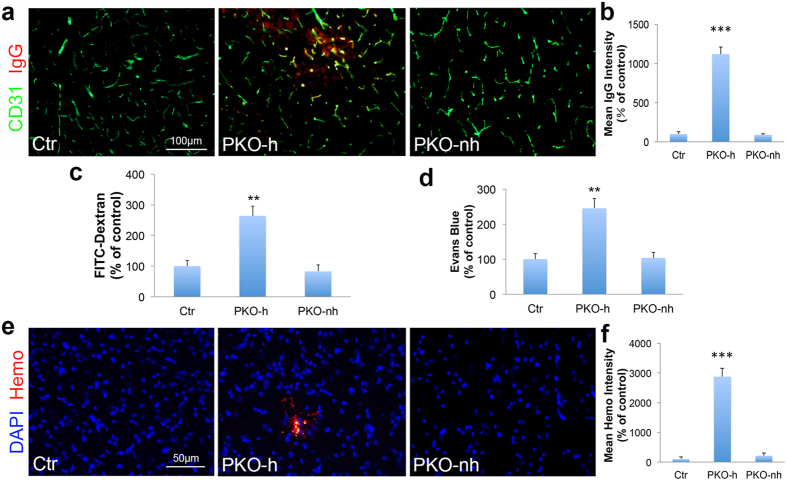
PKO-h mice have BBB breakdown and micro-hemorrhages. (**a**) Confocal images of CD31 (green) and mouse IgG (red) staining in Ctr, PKO-h, and PKO-nh brain parenchyma. (**b**) Quantification of mouse IgG intensity in the brains of these mice. *n* = 5. (**c**) FITC-Dextran levels in the brains of Ctr, PKO-h, and PKO-nh mice. *n* = 6. (**d**) Evans blue levels in the brains of Ctr, PKO-h, and PKO-nh mice. *n* = 6. (**e**) Confocal images of hemoglobin (red) staining in Ctr, PKO-h, and PKO-nh brain parenchyma. (**f**) Quantification of hemoglobin intensity in the brains of these mice. *n* = 5. Ctr, controls; PKO-h, laminin γ1^flox/flox^:Pdgfrβ-Cre^+^ mice with hydrocephalus; PKO-nh, laminin γ1^flox/flox^:Pdgfrβ-Cre^+^ mice without hydrocephalus. Scale bars represent 100 μm in (**a**) and 50 μm in (**e**). **p < 0.01, ***p < 0.001 (Student’s *t*-test).

To evaluate vascular integrity of the PKO mice, we first performed immunohistochemistry against IgG. Although IgG was absent in control and PKO-nh mice at 1–2 months, it was detected in the brains of age-matched PKO-h mice (Fig. 2a). Quantification showed a significantly higher level of IgG in PKO-h brains (Fig. 2b), suggesting breakdown of BBB in these mice. To further test this hypothesis, we injected FITC-Dextran intravenously and Evans blue intraperitoneally into 1–2-month-old control and mutant mice, and examined their leakage into brain parenchyma 12 hours later. Consistent with IgG data, substantially higher levels of FITC-Dextran (Fig. 2c) and Evans blue (Fig. 2d) were found in PKO-h brains, compared to the controls or PKO-nh brains, strongly indicating that the BBB integrity is compromised in PKO-h mice at 1–2 months. Furthermore, we also examined BBB integrity at E15.5 and found no leakage in PKO mice (Supplementary Fig. 1a). Given that the barrier property of BBB is already formed by E15.5^17^,^29^, these results suggest that BBB barrier property formation during early development is not affected by the loss of PDGFRβ^+^ cell-derived laminin. Consistent with the BBB permeability data, we also detected hemoglobin in 1–2-month-old PKO-h but not control or PKO-nh brains (Fig. 2e,f). Altogether, these results suggest that the vascular integrity is indeed disrupted in PKO-h mice.

To further investigate whether BBB disruption in PKO-h mice is due to the lack of pericytic laminin directly or caused by hydrocephalus indirectly, we generated an inducible knockout line by crossing the NG2-CreER^TM^ transgenic mice with our laminin γ1^flox/flox^ mice. We administered tamoxifen into these inducible mice at various developmental stages, and tried to identify and analyze animals that do not develop hydrocephalus. This experiment, however, was not successful due to the long turnover rate of laminin. We treated mice with tamoxifen at either neonatal or embryonic stages consecutively for 5 days, and laminin synthesized at early embryonic stage was still detectable two months after treatment (not shown). These data suggest that the inducible system is unable to ablate laminin function, owing to its extremely long turnover rate.

### Laminin expression is abrogated in PDGFRβ^+^ cells in both PKO-h and PKO-nh mice

**Figure 3:**
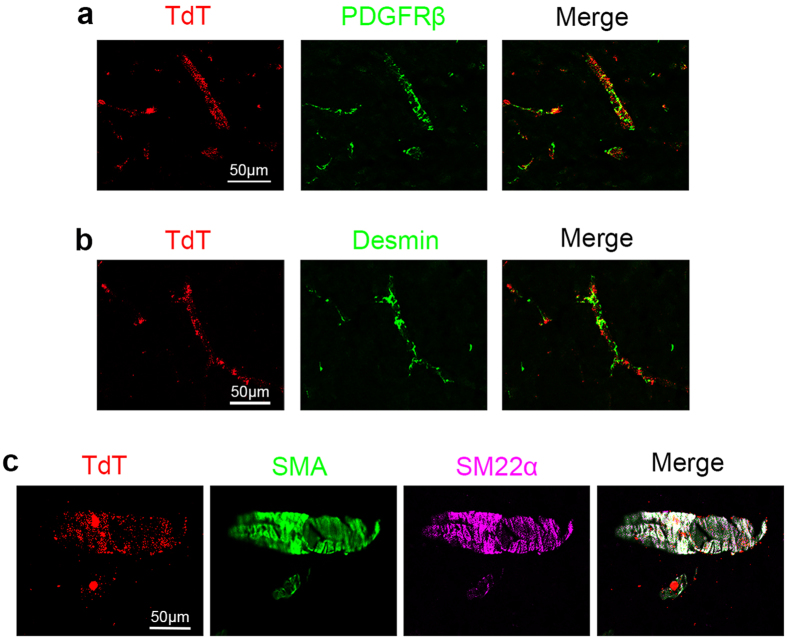
Specificity of Pdgfrβ-driven Cre. Lineage-tracing experiment was performed in Ai14:Pdgfrβ-Cre^+^ reporter mice. TdT (red) expression co-localized with PDGFRβ (green, **a**), pericyte marker Desmin (green, **b**), as well as SMA (green, **c**) and SM22α (magenta, **c**). Ai14, reporter mouse line expressing floxed STOP sequence before TdT; TdT, tdTomato; SMA, smooth muscle actin-α; SM22α, smooth muscle protein 22-α. Scale bars represent 50 μm.

**Figure 4:**
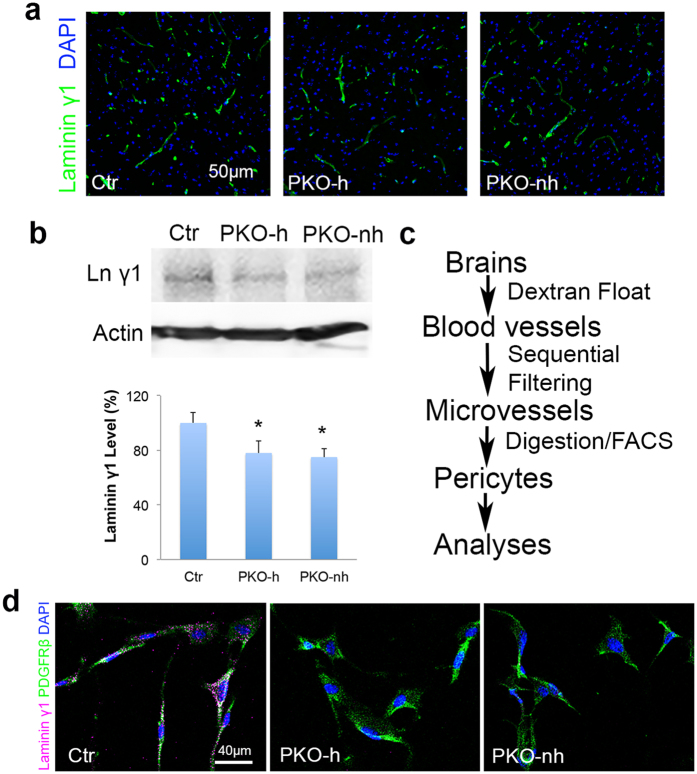
Laminin γ1 expression is abrogated in pericytes in PKO mice. (**a**) Confocal images of laminin γ1 (green) staining in Ctr, PKO-h, and PKO-nh brains. (**b**) Western blot analysis and quantification of laminin γ1 expression in the brains of these mice. *n* = 5. (**c**) Diagram of brain pericyte isolation. (**d**) Immunocytochemistry of laminin γ1 (magenta) and PDGFRβ (green) expression on primary brain pericytes isolated from control, PKO-h, and PKO-nh mice. Ctr, controls; PKO-h, laminin γ1^flox/flox^:Pdgfrβ-Cre^+^ mice with hydrocephalus; PKO-nh, laminin γ1^flox/flox^:Pdgfrβ-Cre^+^ mice without hydrocephalus. Scale bars represent 50 μm in (**a**) and 40 μm in (**d**). *p < 0.05 (Student’s *t*-test).

To determine whether the observed CNS phenotype is due to loss of laminin in PDGFRβ^+^ cells, we first performed lineage-tracing experiments by crossing the Pdgfrβ-Cre^+^ mice with the Ai14 reporter line, which harbors a floxed STOP sequence before reporter gene tdTomato (TdT). In the resulting Ai14:Pdgfrβ-Cre^+^ mice, TdT co-localized with PDGFRβ expression in the brain (Fig. 3a), suggesting that PDGFRβ^+^ cells are indeed targeted for recombination. Next, immunohistochemistry revealed strong laminin γ1 staining in vessels from control brains (Fig. 4a). In both PKO-h and PKO-nh mice, however, laminin γ1 expression was slightly reduced (Fig. 4a). Western blotting revealed slightly but significantly lower levels of laminin γ1 in PKO-h and PKO-nh brains (Fig. 4b), suggesting that laminin expression is decreased in these mice. Furthermore, we isolated PDGFRβ^+^ cells (mostly pericytes due to sequential filtering) directly from control, PKO-h, and PKO-nh brains, and examined their expression of laminin (Fig. 4c). Laminin γ1 expression was found in PDGFRβ^+^ cells isolated from control brains but not in those isolated from PKO-h or PKO-nh brains (Fig. 4d), suggesting that laminin γ1 expression is abrogated in PDGFRβ^+^ cells from both PKO-h and PKO-nh mice. In addition, we also isolated astrocytes and brain microvascular endothelial cells from these mice and examined their laminin expression. Consistent with previous reports^13^,^14^,^15^,^16^, astrocytes predominantly express laminin α1 and α2, whereas brain microvascular endothelial cells mainly produce laminin α4 and α5 (Supplementary Fig. 2). The same expression pattern and comparable expression levels of these laminin α chains were observed in PKO-h and PKO-nh cells (Supplementary Fig. 2), suggesting that loss of PDGFRβ^+^ cell-derived laminin does not affect laminin expression in astrocytes or brain microvascular endothelial cells.

**Figure 5:**
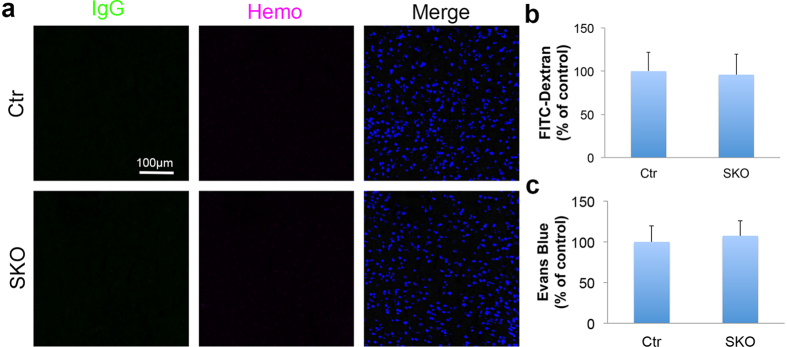
Vascular integrity is unaffected in SKO mice. (**a**) Confocal images of IgG (green) and Hemo (magenta) staining in Ctr and SKO brain parenchyma. (**b**) FITC-Dextran levels in the brains of Ctr and SKO mice. *n* = 5. (**c**) Evans blue levels in the brains of Ctr and SKO mice. *n* = 5. Ctr, controls; SKO, laminin γ1^flox/flox^:Transgelin/SM22α-Cre^+^; Hemo, hemoglobin; FITC, fluorescein isothiocyanate. Scale bar represents 100 μm.

It has been shown that PDGFRβ labels both pericytes and VSMCs^30^. Consistent with these reports, TdT co-localized with both pericyte marker Desmin (Fig. 3b) and VSMC markers SMA and SM22α (Fig. 3c) in our lineage-tracing experiment. To further determine whether the CNS phenotype is due to loss of laminin in pericytes or VSMCs, we also generated a VSMC-specific conditional knockout line (laminin γ1^flox/flox^:Transgelin/SM22α-Cre^+^, termed SKO hereafter) by crossing the laminin γ1^flox/flox^ mice with the Transgelin/SM22α-Cre^+^ line^12^. The specificity of the Transgelin promoter has been validated in a variety of studies^31^,^32^,^33^. The SKO mice are born at expected Mendelian ratio with no gross abnormality, although they have a lower blood pressure^12^. Immunohistochemical analysis revealed negligible levels of IgG and hemoglobin in SKO brains (Fig. 5a). In addition, no differences in FITC-Dextran (Fig. 5b) and Evans blue (Fig. 5c) levels in brain parenchyma were found between SKO and control mice. These data suggest that the CNS phenotype (hydrocephalus and BBB breakdown) observed in PKO-h mice is due to lack of laminin expression in pericytes rather than VSMCs.

### Tight junction protein expression is reduced in PKO-h but not PKO-nh mice

**Figure 6:**
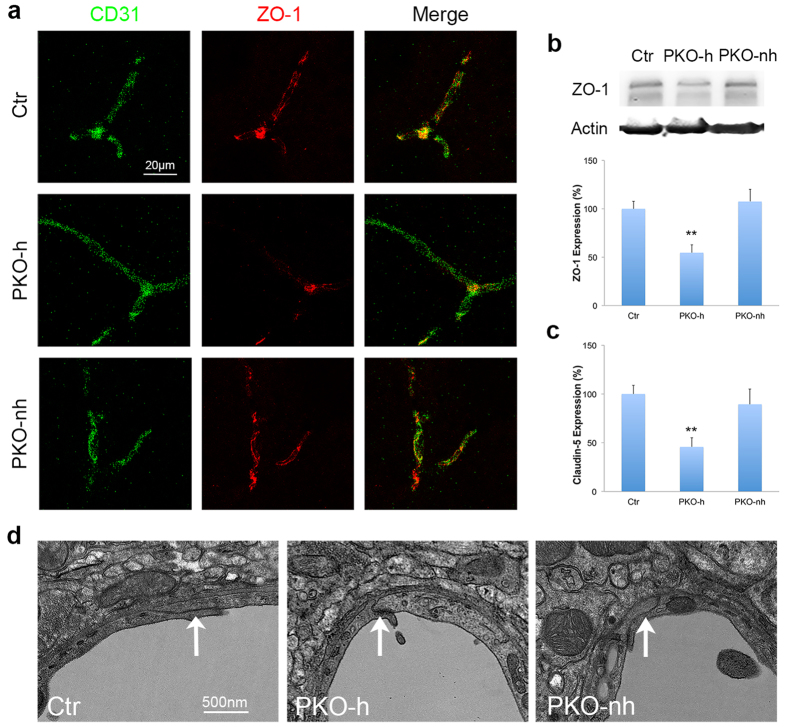
Tight junction proteins are decreased in PKO-h but not PKO-nh mice. (**a**) Confocal images of CD31 (green) and ZO-1 (red) staining in Ctr, PKO-h, and PKO-nh brains. (**b**) Western blot analysis and quantification of ZO-1 expression in the brains of these mice. *n* = 5. (**c**) Quantification of Claudin-5 expression in the brains of these mice. *n* = 5. (**d**) Electron microscopy images of tight junction structure in Ctr, PKO-h, and PKO-nh brains. White arrows indicate tight junctions. Ctr, controls; PKO-h, laminin γ1^flox/flox^:Pdgfrβ-Cre^+^ mice with hydrocephalus; PKO-nh, laminin γ1^flox/flox^:Pdgfrβ-Cre^+^ mice without hydrocephalus. Scale bars represent 20 μm in (**a**) and 500 nm in (**d**). **p < 0.01 (Student’s *t*-test).

To investigate the mechanisms that are responsible for the compromised vascular integrity in PKO-h mice, we first examined tight junction protein expression in endothelial cells. CD31 staining revealed comparable vessel density between control and PKO (both PKO-h and PKO-nh) brains (Fig. 2a), suggesting that the observed abnormalities are less likely due to defects in angiogenesis or differences in vessel density. Next, we examined the expression of tight junction proteins. Consistent with previous data, ZO-1 expression co-localized well with CD31 in 1–2-month-old control mice (Fig. 6a). In age-matched PKO-h mice, however, ZO-1 level was substantially decreased (Fig. 6a). In contrast to the PKO-h mice, PKO-nh mice showed ZO-1 expression to a level comparable to that in control brains (Fig. 6a). Western blot revealed similar results and quantification showed the reduction of ZO-1 in PKO-h brains was statistically significant (Fig. 6b). Like ZO-1, claudin-5 was substantially decreased in PKO-h but not PKO-nh brains (Fig. 6c), suggesting loss of tight junction proteins in PKO-h mice. In addition, we also examined the structure of tight junctions by electron microscopy and found no obvious defects. In control and PKO-nh brains, endothelial cells usually extended stretches that overlapped with each other, forming long junctions (Fig. 6d). In PKO-h brains, however, endothelial stretches tended to grow into each other and short junctions were more frequently observed (Fig. 6d). Altogether, these data suggest that the biochemical components of tight junctions are affected in PKO-h mice.

### Astrocytic expression of AQP4 is reduced in PKO-h but not PKO-nh mice

**Figure 7:**
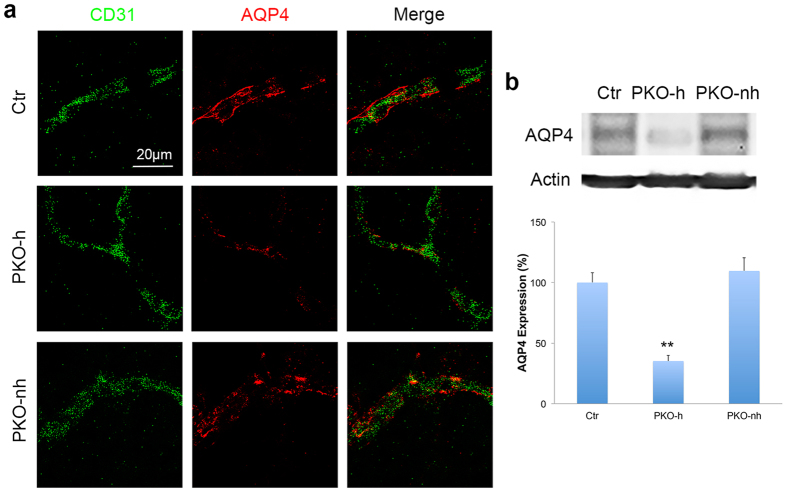
AQP4 expression is reduced in PKO-h but not PKO-nh mice. (**a**) Confocal images of CD31 (green) and AQP4 (red) staining in Ctr, PKO-h, and PKO-nh brains. (**b**) Western blot analysis and quantification of AQP4 expression in the brains of these mice. *n* = 5. Ctr, controls; PKO-h, laminin γ1^flox/flox^:Pdgfrβ-Cre^+^ mice with hydrocephalus; PKO-nh, laminin γ1^flox/flox^:Pdgfrβ-Cre^+^ mice without hydrocephalus. Scale bar represents 20 μm. **p < 0.01 (Student’s *t*-test).

Previous studies have shown that astrocyte polarity, characterized by expression of aquaporin-4 (AQP4) predominantly in endfeet, also contributes to vascular integrity^22^,^34^,^35^,^36^. Therefore, we also examined AQP4 expression in the control and PKO brains. At 1–2 months, AQP4 signal was predominantly found in the abluminal side of CD31^+^ vessels in control mice (Fig. 7a), suggesting that astrocytes are highly polarized. In age-matched PKO-h mice, however, AQP4 expression was significantly reduced and most CD31^+^ vessels were no longer covered by AQP4 (Fig. 7a). In contrast to PKO-h mice, PKO-nh mice demonstrated high level of AQP4 staining (Fig. 7a). Its distribution, however, showed a patchy pattern and was no longer along the abluminal side of CD31^+^ vessels (Fig. 7a). Consistent with these immunohistochemical data, western blotting revealed a significant reduction of AQP4 expression in PKO-h but not PKO-nh brains (Fig. 7b), suggesting that astrocytic expression of AQP4 is severely compromised in PKO-h mice (both expression level and distribution pattern) and moderately affected in PKO-nh mice (only distribution pattern).

### Pericyte density/coverage is altered in PKO-h but not PKO-nh mice

**Figure 8:**
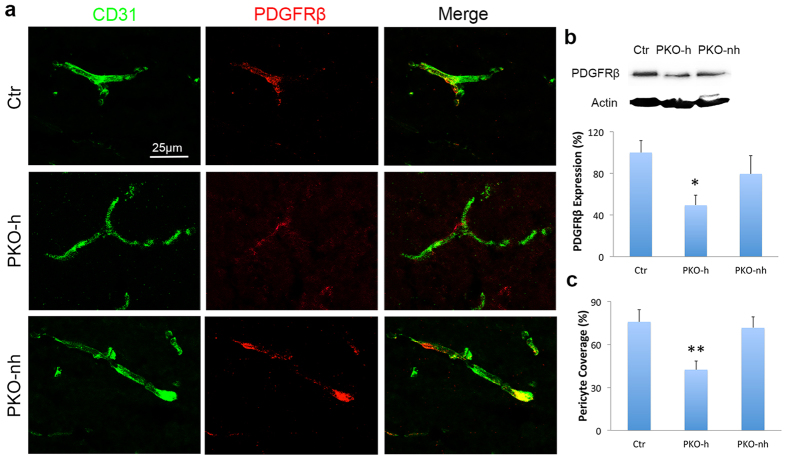
Pericyte coverage is reduced in PKO-h but not PKO-nh mice. (**a**) Confocal images of CD31 (green) and PDGFRβ (red) staining in Ctr, PKO-h, and PKO-nh brains. (**b**) Western blot analysis and quantification of PDGFRβ expression in the brains of these mice. *n* = 5. (**c**) Quantification of pericyte coverage of brain vessels in Ctr, PKO-h, and PKO-nh mice. *n* = 5. Ctr, controls; PKO-h, laminin γ1^flox/flox^:Pdgfrβ-Cre^+^ mice with hydrocephalus; PKO-nh, laminin γ1^flox/flox^:Pdgfrβ-Cre^+^ mice without hydrocephalus. Scale bar represents 25 μm. *p < 0.05; **p < 0.01 (Student’s *t*-test).

Accumulating evidence suggests that pericyte coverage negatively correlates with BBB permeability^17^,^24^,^34^,^37^. To investigate if pericyte coverage is affected in PKO-h mice, we examined PDGFRβ expression by immunohistochemistry. In 1–2-month-old control brains, PDGFRβ co-localized with CD31^+^ vessels, covering most capillaries (Fig. 8a). In age-matched PKO-h brains, however, PDGFRβ expression was dramatically reduced, leaving many CD31^+^ vessels uncovered (Fig. 8a). In PKO-nh mice, PDGFRβ demonstrated an expression pattern similar to that in control (Fig. 8a). Western blot analyses revealed a significant decrease of PDGFRβ intensity in PKO-h but not PKO-nh brains (Fig. 8b). Consistent with these data, pericyte coverage was substantially decreased in PKO-h but not PKO-nh brains (Fig. 8c). Similar result was found when desmin was used as a marker for pericytes in this study (not shown). In addition, similar levels of pericytes were found around CD31^+^ vessels in control and PKO brains at E15.5 (Supplementary Fig. 1b), suggesting that pericyte migration during early development is not affected in the PKO mice.

## Discussion

In this study, we generated the PKO mice by crossing the laminin γ1^flox/flox^ mice with the Pdgfrβ-Cre^+^ line, and categorized them into two groups (PKO-h and PKO-nh) based on the size of their ventricles. We found that PKO-h but not PKO-nh mice showed signs of BBB breakdown and micro-hemorrhage. Consistent with this observation, decreased tight junction proteins, reduced pericyte coverage and compromised astrocyte polarity were detected in PKO-h but not PKO-nh brains. The phenotypes found in PKO-h mice, including hydrocephalus, BBB disruption, and muscular dystrophy, closely mimic the symptoms of dystroglycanopathies, a heterogeneous group of disorders characterized by defective glycosylation of dystroglycan^38^. When properly glycosylated, dystroglycans directly connect the extracellular matrix with cytoskeleton, allowing communications between cells and their environment. Defective glycosylation abolishes appropriate interactions between cells and the extracellular matrix and disrupts tissue organization^39^, leading to muscular dystrophy and brain deformity. Many genes in dystroglycan signaling pathway, including *POMT1*^40^ and *FKTN*^41^, have been found to contribute to the pathogenesis of dystroglycanopathies. Our results suggest that laminin, a ligand for dystroglycan, is involved in the pathogenesis of dystroglycanopathies. This is, to the best of our knowledge, the first direct evidence linking laminin to hydrocephalus.

It should be noted that laminin expression is absent in PDGFRβ^+^ cells from all PKO mice, whereas only 10.7% show brain abnormality (hydrocephalus and BBB breakdown). The absence of brain abnormality in the rest 89.3% of PKO mice could be due to an incomplete penetrance of the CNS phenotype, which can be easily explained by the C57BL/6J × FVB mixed genetic background. Accumulating evidence shows that the genetic background heavily affects the penetrance and/or expressivity of certain phenotypes. For instance, about 0% TGFβ1 null animals survive to birth on a C57BL/6J/Ola background and the number increases to 80% on a NIH/Ola background^42^. Another example is that transgenic mice expressing G93A human SOD1 gene have a more severe phenotype in ALR, NOD.Rag1KO, SJL or C3H background than in B6, B10, BALB/c and DBA lines^43^. In addition, considerable variations in phenotypes due to different genetic background have also been reported in fibronectin^−/−^ and EGFR^−/−^ mice^44^,^45^. Interestingly, although only 10.7% of the PKO mice show brain abnormality (hydrocephalus and BBB disruption), all of them demonstrate a severe muscle deficit^26^. This observation suggests different penetrance for the brain phenotype and muscle phenotype, which may be explained by the fact that brain pericytes and muscle pericytes have distinct biochemical properties and functions. It has been shown that pericytes in the brain and muscle have different developmental origins^24^, which may confer biochemical and functional differences to these two populations and thus result in different penetrance/phenotypes in PKO mice. To test this possibility, Cre lines that specifically target brain pericytes and/or muscle pericytes are required. Unfortunately, such mouse lines are currently not available.

The fact that BBB breakdown always goes hand in hand with hydrocephalus suggests that BBB disruption may be secondary to hydrocephalus. There is evidence showing that impairment of BBB integrity is in association with hydrocephalus^18^,^46^,^47^. For example, 3,6-diaminoacridine hydrochloride^48^ and lanthanum chloride^49^ were able to traverse the endothelial tight junctions and infiltrate into brain parenchyma in hydrocephalic mice. In addition, structural changes in endothelial cells were observed in both hydrocephalic rats^50^ and hydrocephalic human patients^50^,^51^,^52^. Moreover, BBB-related transporters, including P-glycoprotein, were altered in hydrocephalic rats^53^,^54^. However, due to technical difficulties (e.g., long turnover rate of laminin), we are unable to experimentally demonstrate that the BBB breakdown in a small percentage (10.7%) of PKO mice is secondary to or caused by hydrocephalus.

Compared to the phenotype of astrocytic laminin deficiency (BBB breakdown and age-dependent intracerebral hemorrhage)^22^,^23^, loss of pericytic laminin leads to a milder phenotype in vascular integrity. This mild phenotype may be explained by the anatomical structure of the BBB and laminin isoforms expressed by these cells. At the BBB, pericytes are sandwiched between endothelial cells and astrocytic endfeet^24^, and covered by laminin-containing BM synthesized by all three cell types. Previous studies reported that astrocytes produce laminin α1 and α2, whereas endothelial cells make laminin α4 and α5^13^,^14^,^15^,^16^. Our data showed that pericytes, like endothelial cells, predominantly synthesize laminin α4 and α5. Thus, loss of pericytic laminin could be compensated by endothelial laminin, leading to a mild phenotype; while loss of astrocytic laminin can not be compensated by laminin synthesized by endothelial cells or pericytes, resulting in a severe phenotype. This explanation is consistent with the phenotype of laminin α4 deficient mice. The laminin α4^−/−^ mice developed hemorrhage in embryonic and neonatal stages, but were recovered in adulthood^21^. The recovery may be explained by the contribution of pericyte-derived laminin α4. However, since the PKO mice were not in a pure background, we cannot exclude the possibility that the mixed genetic background plays a role in the reported phenotype.

Consistent with the important roles of laminin in vascular integrity regulation, accumulating evidence shows that laminin receptors also actively regulate vascular permeability. For example, binding of laminin to integrin α1β1 and α6β1 promotes endothelial cell differentiation and vessel stabilization^55^. Blocking integrin β1, on the other hand, decreased tight junction protein expression and increased vascular permeability^56^. In addition, deleting either subunit of integrin αvβ8, which can bind laminin, vitronectin, and collagen IV^57^,^58^,^59^, leads to defective vascularization and intracerebral hemorrhage^60^,^61^. Furthermore, conditional knockout of integrin αv in astrocytes or neural progenitors results in severe hemorrhage^62^. Although hemorrhage was absent in mice with integrin β8 deficiency in neural progenitors, BBB breakdown was identified in these mice^63^. Altogether, these data suggest that laminin, by binding to its receptors, contributions to the regulation of vascular integrity. In addition to laminin, other extracellular matrix proteins and their receptors also participate in the regulation of vascular integrity. For example, inactivation of fibulin-1 leads to massive hemorrhage and perinatal lethality^64^. Loss of nidogen-1 results in structural changes in the basement membranes at brain capillaries^65^. Next, recurrent multifocal hemorrhage was identified in Col4a1^+/Δex41^ mice, which generate mutant Col4a1 lacking exon 41 that accumulates within cells^66^. Further studies showed that accumulation of this mutant protein in endothelial cells or pericytes, but not astrocytes leads to hemorrhage^66^. Altogether, these studies strongly suggest that extracellular matrix proteins and their receptors play critical roles in vascular integrity regulation.

## Materials and Methods

### Animals

Laminin γ1^flox/flox^ mice were crossed with the Pdgfrβ-Cre^+^ and Transgelin (SM22α)-Cre^+^ transgenic lines to generate PKO (laminin γ1^flox/flox^:Pdgfrβ-Cre^+^) and SKO (laminin γ1^flox/flox^:Transgelin/SM22α-Cre^+^) mice, respectively. For PKO mice, those with and without hydrocephalus were defined as PKO-h and PKO-nh, respectively. In addition, Laminin γ1^flox/flox^ mice were crossed with NG2-CreER^TM^ to generate inducible knockout mice. For lineage tracing experiment, Ai14 reporter line was crossed with the Pdgfrβ-Cre^+^ line and the resulting Ai14:Pdgfrβ-Cre^+^ mice were used for experiments. The PKO mice used in this study were in a C57BL/6J × FVB mixed background, and both sexes were used. All mice were maintained in the animal facility at the University of Minnesota with free access to water and food. Experimental procedures were in accordance with the NIH guide for care and use of animals and were approved by the Institutional Animal Care and Use Committee at the University of Minnesota.

### Primary Cell Isolation

#### Pericyte

Primary brain pericytes were isolated as described previously with modifications^22^,^67^. Briefly, brains were collected under aseptic conditions and meninges were removed under dissection microscope. Next, the brains were minced with a blade and triturated. The tissue solution was then incubated with 0.1% collagenase at 37 °C for 1 hour, and centrifuged at 700 g for 8 minutes. The pellet was resuspended in 17% sterile dextran solution, followed by centrifugation at 6,000 g for 20 minutes at 4 °C. Blood vessel-containing pellet was washed in DMEM and sequentially filtered through 100 μm and 40 μm cell strainers. Microvessels trapped on 40 μm cell strainer were further digested with 1 mg/ml collagenase/dispase (Roche) for 4 hours with constant shaking at 37 °C. Then, the cells were stained with anti-CD31-APC (Biolegend, 102509), anti-CD45-FITC (Biolegend, 103108), and anti-PDGFRβ-PE (eBioscience, 12-1402), and subjected to FACS. Sorted pericytes (PE^+^ APC^−^FITC^−^) were grown in Pericyte Medium (ScienCell, 1201) and used for immunocytochemistry.

#### Brain Microvascular Endothelial Cells

Brain microvascular endothelial cells were isolated using the same protocol. Briefly, brains were processed similarly as described above. After staining, endothelial cells (PE^−^APC^+^ FITC^−^) were isolated by FACS and grown in endothelial medium.

#### Astrocytes

Primary astrocytes were isolated from adult mouse brains as described previously with minor modifications^68^,^69^. Briefly, brains were collected and minced as described above, followed by incubation with papain (20 U/ml) for 90 minutes at 34 °C. Then protease inhibitor solution was added and the tissue was triturated with 5 ml serological pipettes. After passing through 70 μm cell strainer, the cell suspension was subjected to myelin removal using Myelin Removal Beads II (Miltenyi Biotec, 130-096-733) according to the manufacturer’s instruction. Next, the cell suspension was sequentially added to petri dishes pre-coated with CD45, CD31, O4, and CD90 antibodies to remove microglia/macrophages, blood vessels, OPCs, and neurons, respectively. Cells were allowed 15 minutes at room temperature to attach to each petri dish. Unbound cells were then transferred to the next petri dish. After the last petri dish, unbound cells (astrocytes) were collected and cultured in DMEM supplemented with 10% FBS and 1% Penicillin/Streptomycin.

### Immunohistochemistry and Immunocytochemistry

Brain sections (20 μm) and sorted pericytes were fixed in 4% PFA and immunostained with anti-laminin γ1 (Abcam, AB3297, 1:200; NeoMarkers, RT-795-P0, 1:100), anti-laminin α1 (R&D, MAB4656, 1:200), anti-laminin α2 (Sigma, L0663, 1:200), anti-laminin α4 (Sigma, SAB4501719, 1:200), anti-laminin α5 (Sigma, SAB4501720, 1:200), anti-CD31 (BD Pharmingen, 553370, 1:200), anti-Claudin-5 (Invitrogen, 34-1600, 1:400), anti-ZO-1 (Invitrogen, 61-7300, 1:400), anti-AQP4 (Millipore, AB3594, 1:500), anti-PDGFRβ (eBioscience, 14-1402, 1:100), anti-hemoglobin (Antibodies-online, ABIN1078132, 1:200), anti-NG2 (BD, 554275, 1:200), anti-Desmin (Acris, BM5500P, 1:400), anti-SMA (Sigma, F3777, 1:1000), and anti-SM22α (GeneTex, GTX113561, 1:200) antibodies overnight at 4 °C, followed by fluorescent secondary antibodies (Invitrogen) for 1 hour at room temperature. These antibodies have been validated in previous studies^22^,^23^. Then, 20 μm-thick z-stacks were captured using a LSM 710 confocal microscope. Imaging processing was performed using ImageJ and Adobe Photoshop CC. All immunohistochemistry images are projections of the entire 20 μm-thick sections and all immunocytochemistry images are individual slices. For IgG and Hemo quantification, mean signal intensity per field was used. Sections from control and knockout mice were placed in the same slides and processed in parallel. At least three random images from each section and at least three sections (more than 300 μm apart from each other) per sample were analyzed for this analysis. Five animals were used for quantifications.

### Pericyte Coverage Analysis

Pericyte coverage analysis was performed as described previously^17^,^34^,^70^. Briefly, brain sections were immunostained with CD31 to label blood vessels and PDGFRβ or NG2 or Desmin to label pericytes as described above. The areas covered by these signals were measured by ImageJ (area measurement tool). Pericyte coverage was quantified as the percentage (%) of PDGFRβ^+^ or NG2^+^ or Desmin^+^ pericyte area covering CD31^+^ capillary area per field. At least three random images from each section and five sections (more than 300 μm apart from each other) per sample were analyzed for this analysis. Five animals were used for quantifications.

### Histology

H&E staining was performed according to standard protocols.

### Ventricle Size Quantification

It has been shown that the ventricle-to-brain ratio is linearly related to ventricle size and it provides a measurement of hydrocephalus severity^27^,^28^. Thus, the ventricle-to-brain ratio was quantified and used to reflect the size of ventricles as described previously with minor modifications^27^,^28^. Specifically, brains were subjected to sequential sectioning, and sections at the level of striatum and anterior commissure were selected for quantification. The ventricle-to-brain ratio was obtained by dividing the maximal width of the lateral ventricles with the maximal brain width.

### BBB Permeability Assays

#### For adults

sterile Evans blue solution (2% in saline, 10 μg per g of body weight) or 4 kD-FITC-Dextran (25 mg/ml, 50 μl) was injected intraperitoneally or intravenously into control, PKO-h, PKO-nh, and SKO mice, respectively. Twelve hours later, these mice were transcardially perfused with 50 ml saline, and the brains were removed and divided into two hemispheres. The right hemispheres were homogenized in 800 μl saline followed by sonication and centrifuge (13200 rpm for 20 minutes at 4 °C). For FITC-Dextran, the supernatant was collected and read in a fluorescent plate reader (Biotek) at 485/528 nm. For Evans blue, 400 μl supernatant was collected and mixed with equal amount of 50% trichloroacetic acid, followed by incubation over night at 4 °C. After centrifuge (13200 rpm for 20 minutes at 4 °C), the supernatant was collected and read in a spectrophotometer (Biotek) at 620 nm. Each sample was measured in triplicates and the average of these technical replicates was used as one biological replicate. Six animals per group were used for quantification. The results were normalized to the controls.

#### For P2 pups

P2 pups were anesthetized and 10 μl of 4-kD FITC-Dextran (25 mg/ml) was injected into the left ventricle with a Hamilton syringe. After 5 minutes of circulation, brains were collected and fixed in 4% PFA overnight at 4 °C. The brains were then incubated in 30% sucrose for 24 hours and frozen in OCT. 20 μm-thick sections cut with a cryostat were used for immunohistochemistry.

#### For embryos

Embryonic BBB permeability assay was performed according to a published protocol^71^. Briefly, a Cesarean section was performed on pregnant females. Each E15.5 embryo was injected with 5 μl FITC-Dextran (4 kD, 25 mg/ml) into the liver using a Hamilton syringe, while they were still attached via the umbilical cord to the mother’s circulation. After 3 minutes, the heads were collected and fixed in 4% PFA overnight at 4 °C. The heads were then incubated in 30% sucrose for 24 hours and then frozen in OCT. 20 μm-thick sections cut with a cryostat were used for immunohistochemistry.

### Western Blot

Brain samples were homogenized in lysis buffer on ice. Equal amount of proteins was loaded and separated in SDS-PAGE and then transferred to PVDF membranes (Millipore). The membranes were probed with anti-laminin γ1 (Thermo, RT-795, 1:500), Claudin-5 (Thermo, 35-2500, 1:500), ZO-1 (Thermo, 61-7300, 1:500), AQP4 (Millipore, AB3594, 1:500), PDGFRβ (Cell Signaling, 3169, 1:500), and anti-actin (BD, 612657) antibodies over night at 4 °C. Next, the membranes were incubated with appropriate secondary antibodies at room temperature for 1 hour. Proteins were visualized by ECL (PerkinElmer, NEL104001EA) and ChemiDoc Imaging System (Bio-Rad). The density of bands was quantified using Quantity One and normalized to actin. The expression levels of these proteins in PKO-h and PKO-nh brains were normalized to that in control brains. Five animals per group were used for quantification.

### Electron Microscopy

Electron microscopy was performed as described previously^22^. Briefly, age-matched control, PKO-h, and PKO-nh mice were anesthetized and perfused with PBS followed by fixing buffer (0.1 M sodium cacodylate buffer with 2% paraformaldehyde and 2% glutaraldehyde). Next, the striatum was carefully dissected out and fixed in fixing buffer overnight. The tissue was then post-fixed in 1% osmium tetroxide and 1% K-ferrocyanide, en bloc stained with 2% uranyl acetate, and embedded in resin. Ultra-thin sections were cut on a Reichert-Jung Ultracut E microtome. After post-stained with 2% uranyl acetate and 1% lead citrate, the sections were examined and photographed using JEOL100CXII at 80 KV.

### Statistics

Results are shown as mean ± S.D. Student’s t-test, performed by SPSS Statistics or Microsoft Excel, was used to analyze differences between two groups. Sample number (n) represents biological replicates.

## Additional Information

**How to cite this article**: Gautam, J. *et al.* The role of pericytic laminin in blood brain barrier integrity maintenance. *Sci. Rep.* **6**, 36450; doi: 10.1038/srep36450 (2016).

**Publisher’s note:** Springer Nature remains neutral with regard to jurisdictional claims in published maps and institutional affiliations.

## Supplementary Material

Supplementary Information
