# Supplementary material for: The role of pericytic laminin in blood brain barrier integrity maintenance
Source: Sci Rep. 2016 Nov 3;6:36450. doi: 10.1038/srep36450 (PMC5093438; doi:10.1038/srep36450)
Supplement: Supplementary Information [file srep36450-s1.doc]

**The role of pericytic laminin in blood brain barrier integrity maintenance**

Jyoti Gautam, Xuanming Zhang, Yao Yao*

College of Pharmacy, University of Minnesota, 1110 Kirby Drive, Duluth, MN, 55812

Abbreviated Title: Pericytic laminin and BBB integrity

*Corresponding author:

Yao Yao, PhD

Assistant Professor

College of Pharmacy University of Minnesota

1110 Kirby Drive, Duluth, MN 55812

Phone: 218-726-6082; Email: [yyao@umn.edu](mailto:yyao@umn.edu)


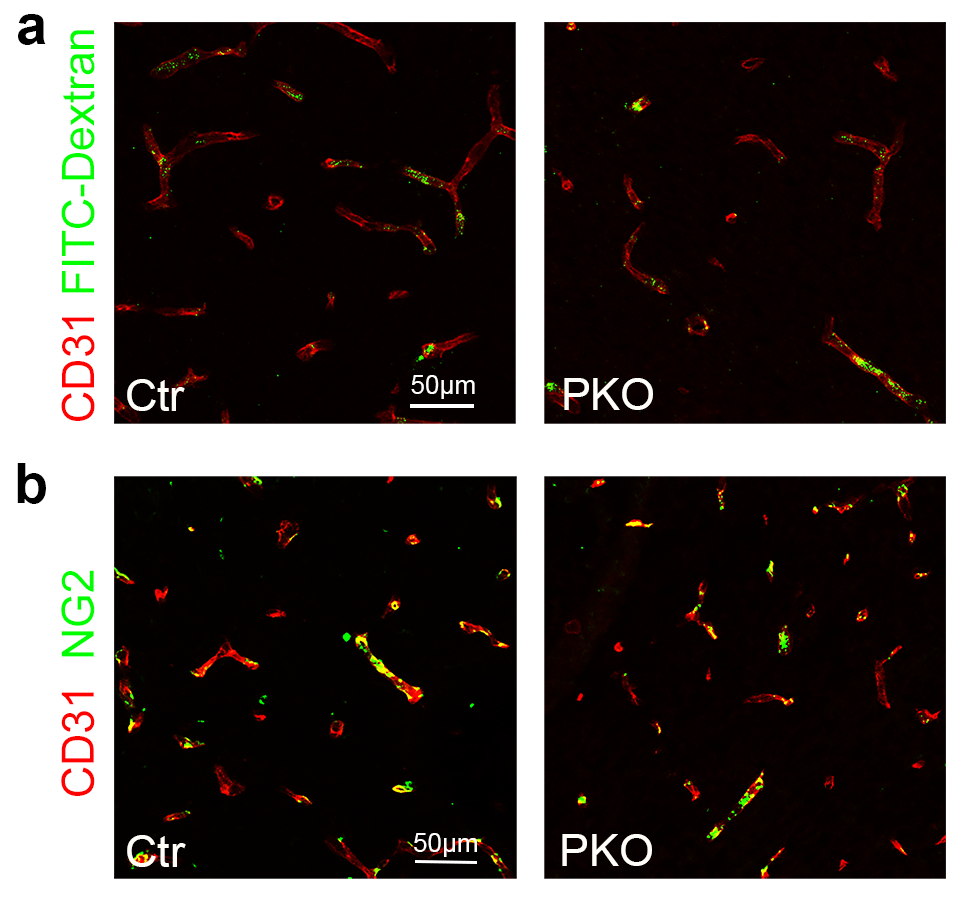


**Supplementary Fig. 1** BBB integrity and pericyte migration are unaffected at E15.5. **a** Confocal images of CD31 (red) and FITC-Dextran (green) staining in Ctr and PKO brain parenchyma at E15.5. **b** Confocal images of CD31 (red) and NG2 (green) staining in Ctr and PKO brain parenchyma at E15.5. Ctr, controls; PKO, laminin γ1^flox/flox^:Pdgfrβ-Cre^+^; BBB, blood brain barrier; E15.5, embryonic day 15.5; FITC, fluorescein isothiocyanate. Scale bars represent 50 μm.


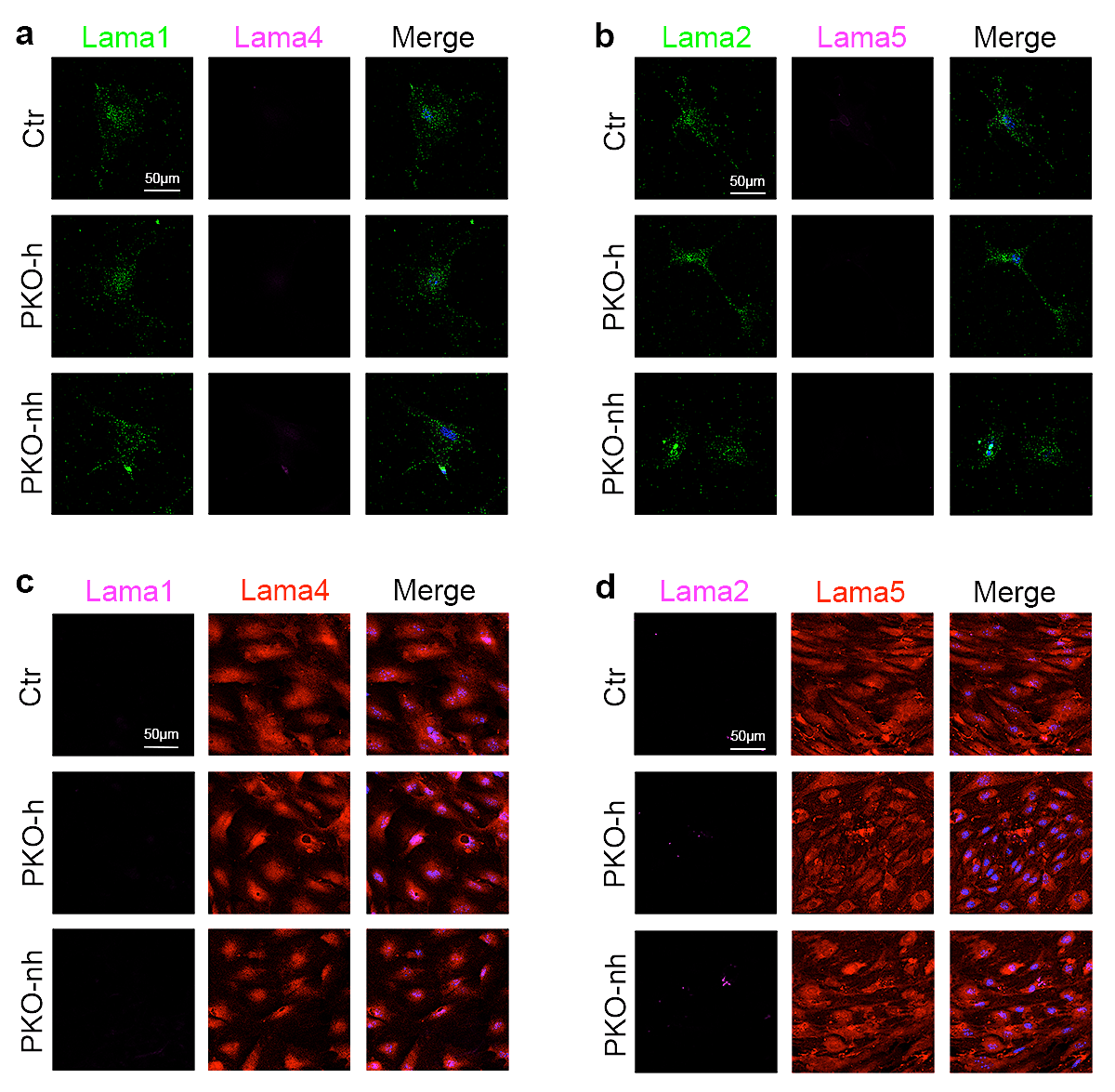


**Supplementary Fig. 2** Laminin expression is not affected in astrocytes and brain microvascular endothelial cells in PKO mice. **a** Immunocytochemistry of laminin α1 (green) and α4 (magenta) expression on primary astrocytes isolated from control, PKO-h, and PKO-nh mice. **b** Immunocytochemistry of laminin α2 (green) and α5 (magenta) expression on primary astrocytes isolated from control, PKO-h, and PKO-nh mice. **c** Immunocytochemistry of laminin α1 (magenta) and α4 (red) expression on primary brain microvascular endothelial cells isolated from control, PKO-h, and PKO-nh mice. **d** Immunocytochemistry of laminin α2 (magenta) and α5 (red) expression on primary brain microvascular endothelial cells isolated from control, PKO-h, and PKO-nh mice. Ctr, controls; PKO, laminin γ1^flox/flox^:Pdgfrβ-Cre^+^; PKO-h, PKO mice with hydrocephalus; PKO-nh, PKO mice without hydrocephalus. Scale bars represent 50 μm.

**Supplementary Table 1** Hydrocephalic rate in control and PKO mice

|  | Control | PKO |
| --- | --- | --- |
| Hydrocephalic (%) | 0.5% | 10.7% |
| Non-hydrocephalic (%) | 99.5% | 89.3% |
